# How to improve aesthetics in patients with Adolescent Idiopathic Scoliosis (AIS): a SPoRT brace treatment according to SOSORT management criteria

**DOI:** 10.1186/1748-7161-4-18

**Published:** 2009-09-01

**Authors:** Fabio Zaina, Stefano Negrini, Claudia Fusco, Salvatore Atanasio

**Affiliations:** 1 ISICO (Italian Scientific Spine Institute), Via Roberto Bellarmino 13/1, 20141 Milan, Italy

## Abstract

**Background:**

Aesthetics is a main goal of both conservative and surgical treatments in adolescent idiopathic scoliosis (AIS). Previously, we developed and validated a clinical scale - the Aesthetic Index (AI)--in order to measure aesthetic impairment and changes during treatment.

**Aim:**

To verify the efficacy of bracing on aesthetics in AIS.

**Study Design:**

Prospective Cohort Study.

**Population:**

Thirty-four consecutive patients, age 13.2 ± 3.7, initial Cobb Angle 32 ± 12°, ATR 10 ± 4° Bunnel, 11 males.

**Methods:**

Patients with AI scores of at least 5/6 were included. Each of them had a brace prescription (18 to 23 hours per day), according to the SPoRT concept. AI was measured again after six months and at the end of treatment, and then the pre- and post-treatment scores compared. The Wilcoxon test was performed.

**Results:**

Twenty-nine patients out of the 34 included completed the treatment and had six-month and final results; four patients were lost during the treatment, and one was fused. At baseline, median AI was 6 (95% IC 5-6) but the score decreased to 3 (95% IC 0-5; p < 0.05) after six months with brace, and this value was maintained in the 29 who completed the treatment (95% IC 1-6; p < 0.05 with respect to the baseline).

**Conclusion:**

Aesthetics can be improved in a clinically significant way when the brace treatment is performed according to the SPoRT concept and by following the SOSORT management criteria. This is a relevant result for patients and a major goal of scoliosis treatment, be it conservative or surgical. The use of a more sensitive tool like TRACE could more easily detect the clinical changes; nevertheless, AI proved sensible enough that its use in everyday clinical practice can be suggested.

## Background

Changes have occurred over the past 20 years in the field of adolescent idiopathic scoliosis (AIS) treatment, so that new outcome measures and new goals in the treatment are gradually emerging. [1] The Cobb angle, despite being the most relevant predictor of worsening during adulthood, is no longer considered a sufficient outcome measure, particularly in the rehabilitative setting. Aesthetic appearance is one of these incoming outcomes: According to a consensus by SOSORT experts, aesthetic improvement has become one of the main goals of scoliosis treatment. [2] Orthopaedic surgeons share the relevance of aesthetics as well: in a recent study concerning the importance of physical deformity of patients with adolescent idiopathic scoliosis, "the severity of deformity" consistently ranked as the most important clinical consideration when proposing surgical treatment to patients. [3]

The results concerning the efficacy of surgical or conservative treatment are frequently discordant. Using the Cosmetic Spinal Score, Theologis et al. found that bracing reduced the rib hump but not enough to improve the aesthetic appearance, while spinal fusion and Harrington instrumentation improved all the measured parameters influencing physical appearance. [4] Contrastingly, Grivas and Vasiliadis showed that a modified Boston brace could improve the aesthetic appearance of the back as measured through the prominence in AIS patients, but more effectively in the double and thoracolumbar curves. [5] Such opposite results can rely or on the specific efficacy of the braces used, or on the different tools employed to evaluate aesthetics. There are many ways to detect and record aesthetic changes -- including questionnaires [6-8], general evaluations of the operator [9] and high-tech instruments [4,10-16] -- but none has been used extensively or achieved any kind of consensus. Our group has more than 20 years' experience in evaluating the aesthetics of the posterior trunk, ranking the asymmetry of the shoulders, scapulae and waist; and over the past five years we have developed and validated a new clinical tool called the Aesthetic Index (AI), which corresponds to the sum of these three subscale scores. [17] Additionally, we have improved this clinical scale by creating and validating the TRACE, (Trunk Aesthetic Clinical Evaluation). According to our clinical experience, braces can modify aesthetics in a rapid way, within the first six months of treatment. The results are then usually maintained if the patient continues his or her treatment, performs proper exercises and completes a gradual weaning process. [18] To check out this clinical hypothesis, we designed the present study to verify the short- and long-term effectiveness of the brace treatment as a means to improve the aesthetic appearance of AIS patients evaluated with a tool specifically designed for that purpose: the Aesthetic Index. We did not use the TRACE because it was not yet developed at the time that patients were treated.

## Methods

We designed a prospective cohort study.

### Population

We included 34 consecutive patients affected by AIS (23 females), age 13.2 ± 3.7 (average ± standard deviation), initial Cobb Angle 32 ± 12°, ATR 10 ± 4° Bunnell (Table 1). The inclusion criteria were: each patient at first evaluation with an AI score of at least 5/6 and a prescription for a brace. The brace was prescribed for a curve of 25° +/- 5 Cobb and Risser 0-3 according to the Italian guidelines on idiopathic scoliosis management. According to patient preferences, there were some exceptions to these rules as follows: patients with angles under 20° but very important aesthetic impact who wished to improve their appearance; patients with Risser 4-5 but important curves who wished to try avoiding surgery.

**Table 1:** pattern of curve at baseline and worst curve magnitude at baseline

Pattern	T + L	TP + T	L	TL	T + TL	T
N° patients	16	1	3	8	2	4

Cobb	32° ± 11	49°	43° ± 19	27° ± 14	31° ± 9	29° ± 4

T + L: Double major Thoracic and Lumbar; TP + T: proximal thoracic + Thoracic; L: Lumbar; TL: Thoracolumbar; T + TL: Thoracic + Thoracolumbar; T: Thoracic

### Treatment protocol

According to the SPoRT concept, at the beginning of treatment each patient had to wear the brace from a minimum of 18 hours to a maximum of 23 hours per day. The family and the patient him/herself were responsible for compliance. During each clinical evaluation they were asked about it by their physician with a series of question.

After a corrective phase, patients were prescribed to gradually reduce the everyday usage but never prescribed fewer than 18 hours per day until Risser 3 stage was attained. At that moment, despite the patient's age, weaning commenced. All the patients gradually reduced the daily hours of brace usage by two to three hours (according to clinical and/or radiographic evaluations) over intervals of six months until the prescription reached eight hours nightly, and then stopped after six months. This weaning phase never requires less than 2 years. The end of brace treatment is at Risser 5 or one year after Risser 5. [18] according to individual needs. Patients performed specific exercises according to SEAS protocol throughout the treatment. [19] All the braces were built according to the SPoRT concept, [20] which is the acronym of Symmetric, Patient-oriented, Rigid, Three-dimensional, active. The SPoRT concept always requires a customised construction of the brace according to the patient's individual requirements. It's possible to apply CAD-CAM technologies, which usually allow us to obtain the best results in this case, but without using pre-built forms stored in databases, as is usually done. Once done, a final test must be made on the patient so as to change the first theoretical project and adapt it in the best possible way, depending on the real interaction between the body and the brace.

The treatment was performed according to SOSORT criteria. [21]

### Procedures and data analysis

AI is a clinical three point scale for asymmetry (0 absent, 1 slight, 2 important asymmetry) of the shoulders, scapulae and waist; AI is given by the sum of these sub-scores, where 6 points is the worst aesthetic situation (highest rate of asymmetry) and 0 is the best (no asymmetries)[17] AI was recorded by an expert operator (SN) at the baseline, after six months and after the end of treatment, and pre and post median scores were compared. The measures were recorded during each single clinical evaluation in a blinded way with respect to the previous ones, by looking directly at the back of patients. No photographs were used and all the records were performed by the same treating physician for each single patient. We also analysed the number of patients who were clinically improved to a significant level, setting the cut-off at three points for AI (the minimum significant change according to our study on the repeatability of AI). [17]

The Wilcoxon test was performed for the pre- and post-analysis of AI values, and the chi square test was performed to verify differences between improved and stable patients. Moreover, ANOVA was performed for Cobb angles and Bunnell degrees.

## Results

**Figure 1:**
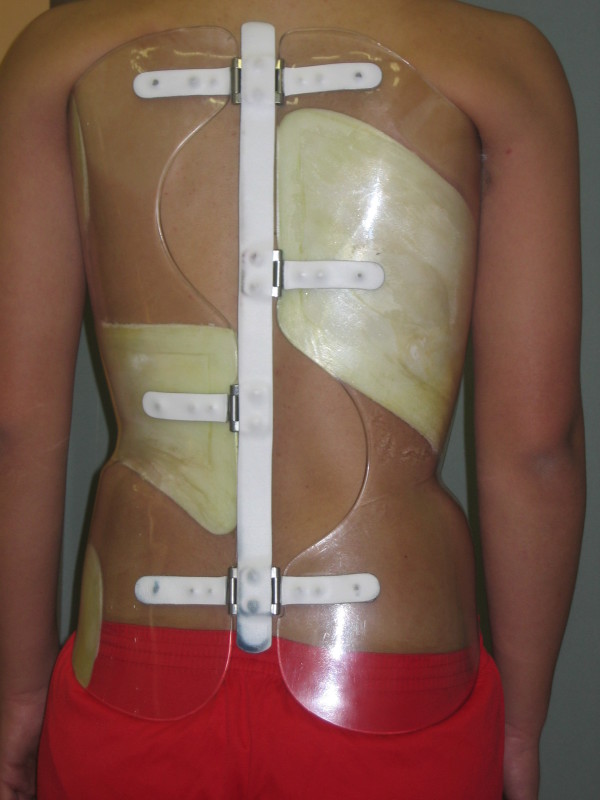
**The Sforzesco brace used by the patients from Fig 4**.

**Figure 2:**
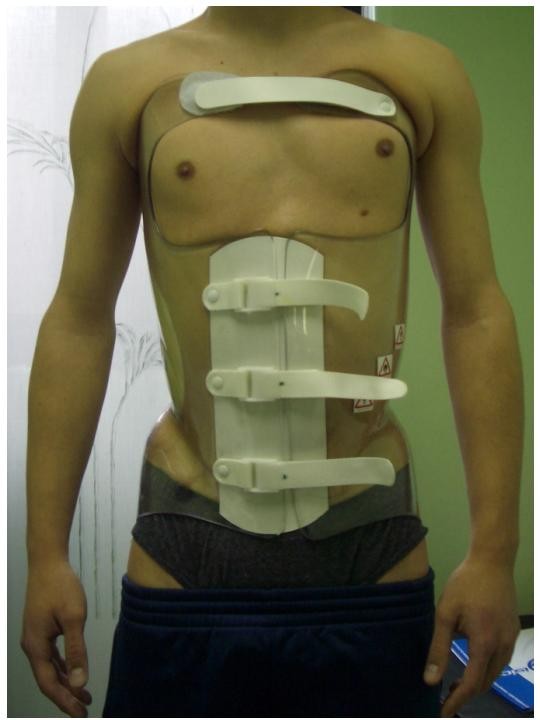
**A frontal view of the Sforzesco brace**.

**Figure 3:**
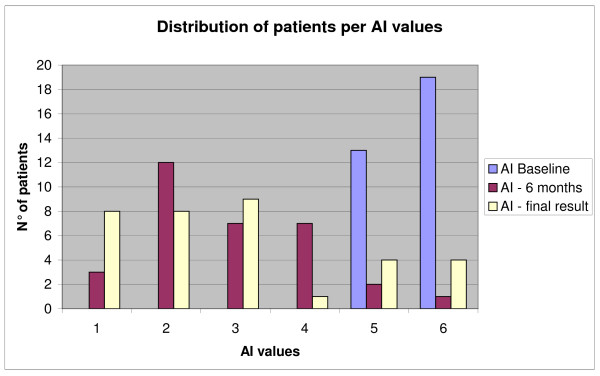
**Distribution of patients according to AI values at baseline, after 6 months and final results**.

**Figure 4:**
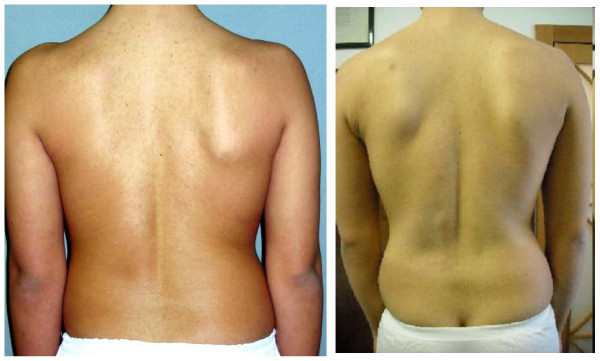
**An example of pre-post treatment results**: on the left a patient at baseline (AI score 5: 2 points waist, 2 points scapulae, 1 point shoulders), on the right the final result (AI score 1: 0 points waist, 1 points scapulae, 0 point shoulders).

Twenty-nine patients out of the 34 included completed the treatment and had both six months and final results; four patients were lost during the treatment, and one was fused. The mean duration of the treatment was 2.5 years (SD 2.1). At baseline, the median AI was a score of 6 (95% IC 5-6), after six months of brace treatment the AI score decreased to 3 (95% IC 0-5; p < 0.05), and this value was maintained in the 29 who completed the treatment (95% IC 1-6; p < 0.05 with respect to the baseline). At six months, all but one patient had improved by at least one point (Fig 1, 2, 3, 4). No differences were detected from 6 months evaluation and the final result.

**Figure 5:**
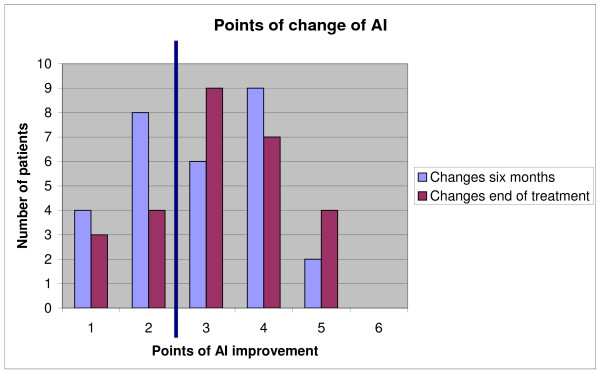
**Points of improvement after six months and at the end of treatment**. The blue line separates the clinically significant results on the right (3 point or more).

Considering the number of patients who were clinically improved at a significant level (three or more points), we found that 56% of the patients had improved at six months and 62% had improved by the end of treatment (Fig 5). Concerning dropouts, the results were not statistically different either at six months or the moment they left the treatment (by which 40% showed improvement).

We tried to make some subgroups for a more precise analysis (subgrouping considering menarche, sex, Risser sign at the beginning) but we found no differences between subgroups.

The mean Cobb angle of the worst curve was 32° ± 12 (range 11-57) at the baseline, and improved to 25° ± 10 (range 5-65) at six months (p < 0.05) and the final result was 28° ± 10 (range 5-49) (p < 0.05). For ATR, there was an improvement from 11° ± 4 to 6° ± 4 (p < 0.05) at six months and a final result of 9° ± 4 (p < 0.05). We found no correlation between the improvement of AI and the initial Cobb angle or the ATR.

## Discussion

Aesthetic improvement is one of the most relevant goals of scoliosis treatment, be it conservative or surgical[2] Braces built according to the SPoRT concept [20] within a treatment regimen based on SOSORT management criteria can achieve this goal. [21]

We had documented, in a previous study, the efficacy of the SPoRT brace in improving aesthetics in AIS scoliosis, with the results being similar to the Risser cast after 18 months of treatment. [22] However, the final results were not yet available as of the beginning of the subject study. Accordingly, this is the first study to document both immediate (six months) and end-treatment aesthetic improvements using a no-cost clinical tool specifically created for this kind of evaluation, while some previous studies used high tech instruments. [16] The main results were achieved within the first six months of treatment, and were maintained until the end of treatment. This issue can be very relevant for the prosecution of treatment, given that a significant trunk remodelling can improve compliance by making the patient more committed to what she or he is doing. The majority of scoliosis treatment outcomes are long-term; nevertheless, such results can seem irrelevant to a young patient without pain and the real perception of a health problem who is asked to wear a rigid plastic piece in order to avoid the risk of back pain in the remote future, along with the worsening of x-ray exams and respiratory problems. On the contrary, such a patient is usually aware of the marked asymmetries of her trunk and wants to improve this aspect as soon as possible. Therefore, the immediate improvement of aesthetics can be of additional help in achieving the improvement of functional outcomes.

Other authors have documented the efficacy of braces to model the trunk deformities in AIS. Grivas interpreted the reduction of prominence as a relevant aesthetic improvement. [5] Koch showed that 73% of AIS patients undergoing surgery were satisfied by the aesthetic result, and that shoulder balance was statistically related to this aspect. [23] Buchanan reported that some surgeons noticed deformities pertaining to the side bending of the trunk and lateral shift. All these parameters are considered in the AI, since they represent the single items on which this scale is built. Moreover, in TRACE we have added the item "hemithorax" as it relates to prominence, [17] which we know from Grivas to be very relevant in some cases. [5]

A previous study showed that AI was a tool of relatively low sensibility, and therefore we developed TRACE which is based on four sub-scales: shoulders, scapulae and waist (which were already present in the AI), and the hemi-thorax. However, the scores for each sub-scale were changed with respect to AI: shoulders now ranged from 0-3, waist from 0-4, scapulae from 0-2 and hemi-thorax from 0-2. From these sub-scales we calculated TRACE, using the sum of the sub-scale scores to reach a 12-point scale. These changes were based on our experience in using the AI. We could not use the latter because the data were collected prior to its development. [17] However, given the fact that TRACE is more sensitive, we can argue that more patients than 62% of those included have reached a clinically significant result with brace treatment.

The measures were recorded during each single clinical evaluation in a blinded way with respect to the previous ones, by looking directly at the back of patients. No photographs were used and all the records were performed by the same treating physician for each single patient. This can seem a limitation of the study, but in our opinion it is a strength. AI demonstrated an intra-observer reliability higher than the inter-observer one, so a single physician can guarantee the maximum sensitivity to changes. Moreover, being this a clinical practice study, the evaluation was made to help the clinical and therapeutic choice and not for research purposes. Finally, at the time when the measures used in this study had been taken, we did not yet consider them as a measurement tool but mainly as an indicator of the aesthetics of the patient to be used in clinical everyday practice for general purposes. All these are guarantees of the honest evaluation by the authors.

The main limitations of this study are represented by the small population included. In the future it will be useful to enlarge the population in order to look for subgroups by which to understand the factors related to aesthetic improvement. We tried a subgroup analysis but we found no differences with regards to sex, Risser sign. The population group on the base of menarche was not homogeneous and the analysis was not performed. Moreover, both AI and TRACE are only clinical scales: This can diffuse their usage during clinical practice, but they have nearly the same low sensitivity to changes. It is possible that some improvement relevant for patients was missed because of this, and for that reason new instruments are being developed and will be available and ready for testing. [24]

Another possible limitation is the lack of a control group. But we can say, based on natural history studies that a spontaneous improvement of the back deformities is almost unlikely to happen. [25]

The main strength of the paper is to be the first to document the aesthetic improvements of a brace treatment for AIS using a specific tool. These results can be achieved quite rapidly, during the first six months of treatment, giving the patient an immediate feedback and probably more motivation to continue the treatment regimen.

## Conclusion

The aesthetic aspect can be improved in a clinically significant way through a brace treatment performed according to the SOSORT management criteria. This is a relevant result for patients and a major goal of scoliosis treatment, be it conservative or surgical. The use of a more sensitive tool such as TRACE could more easily detect the clinical changes, but AI has been shown to be sufficient sensitive and effective to suggest its use in everyday clinical practice.

## Consent

Written informed consent was obtained from the patient for publication of this case report and accompanying images. A copy of the written consent is available for review by the Editor-in-Chief of this journal

## Competing interests

The authors declare that they have no competing interests.

## Authors' contributions

All authors made substantial contributions to conception, design and acquisition of data; they have been involved in drafting and revising the manuscript; they have given final approval of the version to be published.
